# Infection-induced behavioural changes reduce connectivity and the potential for disease spread in wild mice contact networks

**DOI:** 10.1038/srep31790

**Published:** 2016-08-22

**Authors:** Patricia C. Lopes, Per Block, Barbara König

**Affiliations:** 1 Department of Evolutionary Biology and Environmental Studies, University of Zurich, Zurich, Switzerland; 2 Department of Humanities, Social and Political Sciences, ETH, Zurich, Switzerland

## Abstract

Infection may modify the behaviour of the host and of its conspecifics in a group, potentially altering social connectivity. Because many infectious diseases are transmitted through social contact, social connectivity changes can impact transmission dynamics. Previous approaches to understanding disease transmission dynamics in wild populations were limited in their ability to disentangle different factors that determine the outcome of disease outbreaks. Here we ask how social connectivity is affected by infection and how this relationship impacts disease transmission dynamics. We experimentally manipulated disease status of wild house mice using an immune challenge and monitored social interactions within this free-living population before and after manipulation using automated tracking. The immune-challenged animals showed reduced connectivity to their social groups, which happened as a function of their own behaviour, rather than through conspecific avoidance. We incorporated these disease-induced changes of social connectivity among individuals into models of disease outbreaks over the empirically-derived networks. The models revealed that changes in host behaviour frequently resulted in the disease being contained to very few animals, as opposed to becoming widespread. Our results highlight the importance of considering the role that behavioural alterations during infection can have on social dynamics when evaluating the potential for disease outbreaks.

Infection may modify host behaviour through pathogen-dependent^1^ or host-dependent processes^2^. When animals become sick, their behavioural and physiological responses can enable other animals to identify infected individuals and consequentially alter their own behaviours towards them^3^,^4^,^5^,^6^. Together, these factors can alter the dynamics of animal groups when a disease emerges. Rapid changes in the contact network can subsequently alter the speed and magnitude of the disease spread.

Models of disease dynamics have traditionally assumed homogeneity of social contacts within a population^7^. A more biologically relevant approach, however, would be one that encompasses the complex contact patterns experienced by natural populations. By accounting for contact heterogeneity, contact network models have provided valuable insights into the relationship between socio-ecology and disease dynamics^8^,^9^,^10^. Whereas much information has been gained on heterogeneity in human contact patterns^8^,^11^,^12^,^13^, describing contact patterns in free-ranging wildlife populations has only been possible on a few study systems^14^. Yet, because a large percentage of emerging infectious diseases threatening humans originate in wildlife^15^ and diseases in animals can have important impacts on food availability and conservation efforts, studying disease spread in non-human animals is of major importance.

Technological and analytical advances are now facilitating our capacity to map the social networks of animals in the wild^16^. This has made it possible to go from pure modelling studies to studies that use known networks of animals to simulate disease outbreaks^17^,^18^ and to explain patterns of infectious status^19^,^20^,^21^,^22^,^23^. These approaches, however, are limited in their ability to disentangle different factors that determine the outcome of disease outbreaks.

Experimental approaches, integrated with observational and modelling ones, are now critically needed to understand how disease, by altering behaviour, impacts interactions among individuals and thus changes disease transmission dynamics. To quantify the effect of an infection on the dynamics of social contact networks of wild house mice (*Mus musculus domesticus*), we used a common model of bacterial infection, consisting of injections of a bacterial product (lipopolysaccharide or LPS) known to elicit innate immune responses in animals^24^. This immune activation is linked to nonspecific symptoms of infection, including a generalized change in behaviours characterized by decreased activity^2^. Mice in our population spend a considerable amount of time in nests, in contact with other mice (conspecifics). A reduction in activity could increase or decrease transmission by changing patterns of nest use not only by the affected animals (e.g. increased time spent inside nests leads to increased contacts and transmission), but also by conspecifics (e.g. avoidance of nests containing sick animals leads to decreased contacts and transmission). For instance, it is known that rodents can use different sets of cues to avoid sick conspecifics^25^,^26^. Here, we monitored nest-sharing contact networks of wild mice pre- and post-immune challenge and then used the observed changes to model disease transmission within the contact networks.

## Results

### Social groups of wild mice living in a barn

Over 90% of free-living adult mice (257 individuals) inhabiting a barn were implanted with transponder tags, which were read by antennas in 40 artificial nest boxes available for mouse use. Survival and reproductive success in house mice is linked to access to safe nests^27^,^28^. Despite extensive search efforts, we rarely find litters outside of the artificial nest boxes. Even during winter, when reproductive output is low, the mice in the population use the nest boxes extensively. In a single day in winter, we detected up to 26 mice using the same nest box and mice spending up to 20 h inside these boxes. Thus, by providing a space where several mice can be in close contact for extended periods of time, nests form a potentially important medium for transmission of pathogens and disease.

**Figure 1:**
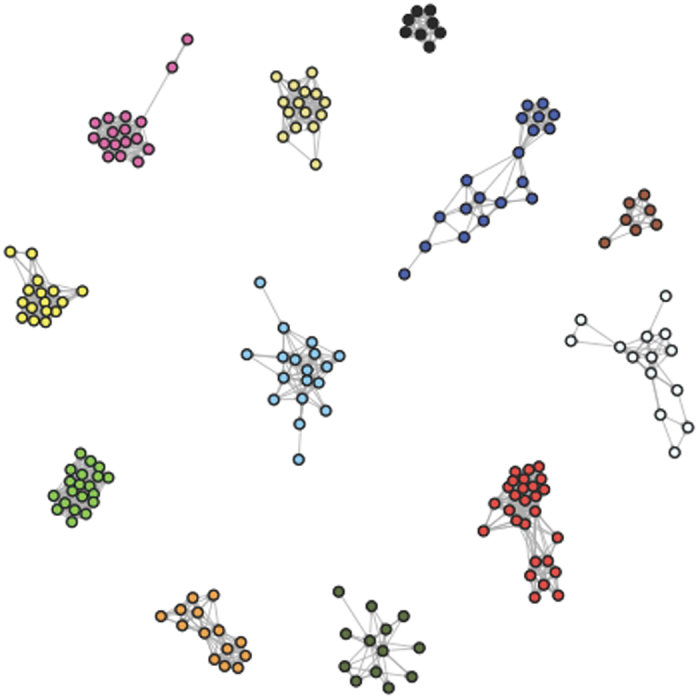
Network representation of social groups of wild mice, obtained using nest box association data over one night. Mice are illustrated as circles and the interactions among them are represented as lines. Different social groups have different colours.

During the experiment, we visited the population every other day, a few hours prior to dusk. Overnight antenna data obtained from the undisturbed days revealed that the population formed 11–12 unconnected social groups (Fig. 1). All of these groups were used, but in a staggered fashion as follows: during each visit, we targeted 3 to 4 social groups, with only one mouse per group receiving an injection of either LPS or control (saline) in one night. Over several weeks, we repeated all social groups, using different animals each time, resulting in 35 control (19 females and 16 males) and 37 LPS (17 females and 20 males) injected animals. We then used data collected by the antennas to quantify treatment differences in individual and social behaviours overnight.

### Reduction of activity in immune-challenged mice

**Figure 2:**
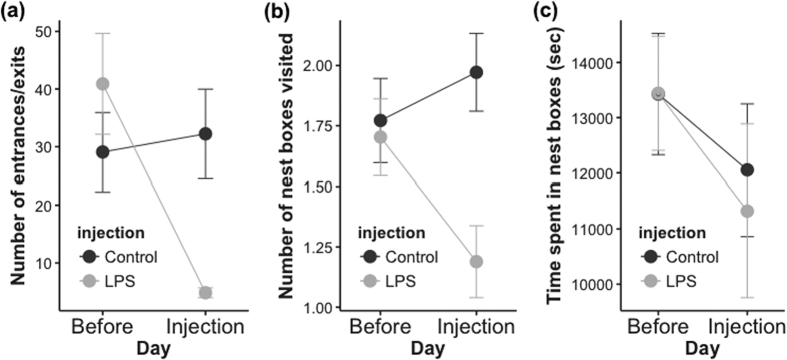
Individual behaviour of mice injected with either LPS or control on the night of injection or two nights before, assessed by nest box use overnight. Data represent means ± SE. (**a**) Number of times animals went in and out of nest boxes; (**b**) Number of nest boxes visited; (**c**) Time animals spent inside nest boxes.

Mice injected with LPS showed reduced movement compared to controls. Specifically, they significantly reduced the number of entrances and exits to nest boxes during the night of injection relative to a previous night (χ^2^ = 10.30, *P* = 0.0013, d.f. = 1; Fig. 2a; no significant effect of sex, χ^2^ = 0.0069, *P* = 0.93, d.f. = 1; parameter estimate ± standard error [SE] = −35.6 ± 10.66 for females and −36.6 ± 10.12 for males in LPS treatment, and 3.71 ± 10.28 for females and 2.69 ± 10.87 for males in control treatment). Moreover, LPS injection led to a significant reduction in number of nest boxes used (Fig. 2b) that differed based on sex (χ^2^ = 4.62, *P* = 0.032, d.f. = 1, for interaction of sex by injection). Because prior to injection females visited on average more boxes than males (2.3 ± 0.18 for females vs. 1.13 ± 0.06 for males), the reduction in nest boxes used due to LPS injection was stronger in females (−1 ± 0.22) than in males (−0.1 ± 0.21). In control-injected animals, no such reduction was generally observed (0.21 ± 0.21 for females and 0.18 ± 0.23 for males). Average time spent inside nest boxes each night was not significantly affected by treatment (χ^2^ = 0.14, *P* = 0.71, d.f. = 1; Fig. 2c) or sex (χ^2^ = 0.0002, *P* = 0.99, d.f. = 1).

### Reduction of interactions with conspecifics

By moving less, mice subsequently came into contact with fewer conspecifics. We constructed a social contact network based on the overlap in time between two or more mice sharing a nest box. We compared the change in social contacts occurring over two nights (prior to injection and during injection) in injected animals to changes occurring in non-injected ones, to account for baseline fluctuations in social interactions. Mice had a significant reduction in number of nest box partners (degree), after being injected with LPS (*P* = 0.008), but not when injected with saline (*P* = 0.19; Table 1). With the antenna setup that we used, we were able to determine which mouse arrived first inside a nest. This knowledge permitted us to distinguish between focal mice being visited and focal mice visiting others. The reduction in degree was driven primarily by the LPS-injected mice visiting a reduced number of animals (average ∆_out-degree_ = −3.2), rather than fewer mice in the network visiting the LPS-injected mice (average ∆_in-degree_ = −1.6; Table 1). Similar patterns were found for time in contact and number of interactions with others (Table 1). Control mice did not demonstrate significant changes for any of these variables (Table 1).

**Figure 3:**
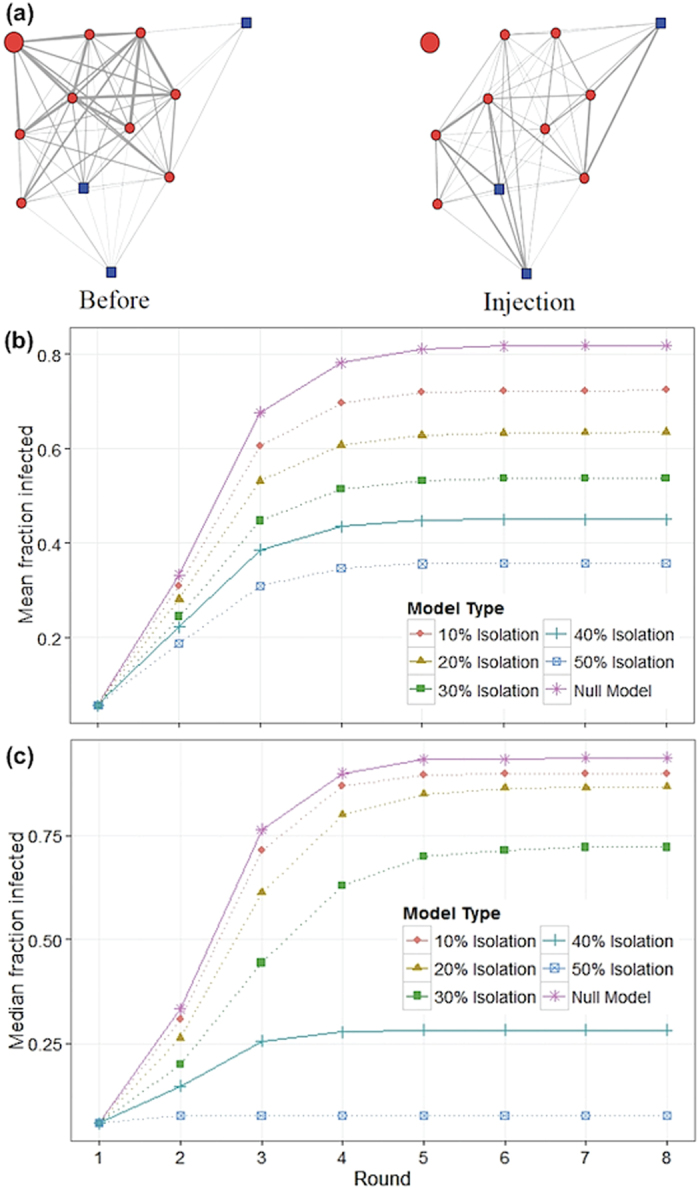
Impact of individual behaviour on transmission. (**a**) Example of network on two separate nights (before and injection night) where one mouse was injected with LPS (larger circle). Males are circles coloured in red, females are squares in blue. The weight of the lines indicates the strength of the association, which is a function of time spent together in the nest box. (**b**) Predicted spread of a disease over the empirically obtained social networks of mice assuming either no change in behaviour of infected animals (“null model”, top line) or changes in the behaviour of 10%, 20%, 30%, 40%, and 50% of infected animals (lower lines). Lines for the “null model” and empirically observed value are solid; lines for the hypothetical values of behaviour change are dotted. Simulation results based on the epidemiological model described in the text for the mean fraction of infected animals by time step. (**c**) Equal to (**b**) with median value plotted.

Changes in number of social partners (degree) were not uniform across all animals. They were largely driven by 12 out of 30 (40%) LPS-injected mice having a significant drop in degree (Fig. S1), with most becoming completely disconnected from their social groups during the night of infection (for example, see Fig. 3a). For the remaining 60%, there was no significant change in degree between the two nights. Distribution of mice in either of the two groups was independent of sex or weight.

### Robustness of social groups

While the social contacts with the LPS-injected animal were reduced, having an LPS-injected animal in the social group did not significantly change the relationships amongst non-injected animals (network density, stability, and average shortest path length of the networks excluding the focal mice showed no significant change after injection with LPS, *P* = 0.8, 0.92 and 0.82, respectively; details for this analysis in Methods).

### Impact of Behavioural Changes on Disease Transmission Dynamics

We employed a simulation model to examine the effect of behavioural changes on disease transmission using the empirically derived networks of non-injected communities of mice in the barn. Our approach included 1) a “null model” that has no behavioural changes and where the disease could spread through all observed network contacts (described below) and 2) a “behavioural model” identical to the null, but where the empirically observed behavioural changes after infection were incorporated. The “null model” started with one randomly infected animal. In the first round, this animal could transmit the disease to animals to which it was directly connected with a probability proportional to amount of time spent together (edge weight, see Fig. 3a). In subsequent rounds, the newly infected animals could spread the disease to their contacts in a similar way. After one round of infection, the infected animals were no longer contagious or susceptible to infection (in Susceptible-Infected-Recovered - SIR - terms, these animals would be considered R). The model was run until all animals were in either state S or R. In the “behavioural model”, we added a rule where every time an animal became infected, it had a fixed chance of becoming isolated from the network, as was empirically observed with our LPS-injected mice. Apart from the observed 40% chance of becoming isolated from the group, we tested the outcome if the probability of isolation was 10%, 20%, 30%, and 50%. For all models, we calculated the fraction of animals infected per round, as well as the cumulative fraction of animals that were infected at any point, in an attempt to understand how the behavioural changes alter the spread of a hypothetical contagious disease.

Our model predicts that the rate of transmission is drastically reduced when infected animals change their behaviour and that the fraction of infected animals never reaches the same levels as in the “null model” (Fig. 3b). Even a 10% isolation probability already had a noticeable effect on transmission (mean fraction of infected animals after 8 rounds was 0.82 for “null model” and 0.72 for 10% isolation). For the observed 40% isolation, the effect was considerable (0.45 mean fraction of infected animals). Moreover, similar to the decrease in average number of social partners (degree), the infection rate was not universally reduced, but instead the higher probability of isolation mainly led to a large proportion of networks where only one or two mice were infected. As soon as disease spreads to more than that, infection of the entire group was highly likely, regardless of isolation probability. This can be represented by the median fraction of infected animals for the different isolation models (Fig. 3c). For an isolation probability of 30% the majority of groups suffered a complete spread of disease with all mice being infected (median proportion of infected animals after 8 rounds was 0.94 for “null model” and 0.72 for 30% isolation). However, for an isolation probability of 40%, spread of the disease was contained to a few animals in the majority of groups (0.28 median proportion of infected animals).

## Discussion

Previous research on disease spread in wild populations has frequently assumed that social contacts remain unaltered as a disease invades the population. Through a novel combination of experimental manipulations of free-living house mice belonging to a long-term population study, remote tracking, social network analysis and disease modelling, we show here that this is not the case. We found that the behavioural response to an inflammatory challenge can lead to very localized changes of social connectivity that have a large impact on disease transmission within social groups.

Our data suggest that immune-challenged mice became disconnected from their social groups as a result of their own behaviour, rather than through avoidance by conspecifics. We found that immune-challenged animals reduced their movement as quantified by the number of times they entered and exited nest boxes, which impacted the number of different nest boxes used over night. Despite being able to detect diseased and immune-challenged conspecifics^26^,^29^, mice in this study did not reduce the number of visits paid to the immune-challenged mice. Several lines of evidence indicate that urinary cues aid in the detection of diseased conspecifics in mice^29^. In fact, we have shown that mice derived from this wild population exhibit changes in levels of urinary cues when injected with LPS and that females are able to distinguish between an LPS and a saline injected male, preferring to spend time near the latter^26^. It is possible that the urinary cues that allow for this discrimination are absent or reduced inside the nest and, thus, when diseased animals remain in the nest it may be harder to detect them through use of these types of cues. Avoidance of diseased conspecifics has been found in many taxa (for example, lobsters^3^, frogs^30^, fish^31^,^32^, birds^33^) but it is not a rule. For example, banded mongooses with clinical (including behavioural) signs of tuberculosis were not avoided by conspecifics^34^. House finches demonstrated a preference for feeding near conspecifics infected with visible signs of transmissible conjunctivitis^4^. Thus, another possibility for the lack of avoidance in the current study is that even if mice were able to detect diseased conspecifics, they did not adjust their behaviours towards them. This was recently demonstrated by Zala and colleagues^35^, whereby female mice did not avoid mating with males infected with *Salmonella enterica* despite being able to distinguish amongst, and being given a choice between, infected and non-infected males.

While on average we observed a decrease in the number of interactions (degree) of LPS injected animals with conspecifics, we found that these changes were strongly driven by 40% of all the LPS injected mice, which became entirely disconnected from their social groups. The fact that these changes were not uniform could indicate that the inflammatory challenge interacted with other factors (such as social status) in determining the extent to which behaviours changed^36^.

It is interesting to note that changes in social connectivity were driven by and mainly localized around the infected individual, while the network of the rest of the group remained largely unaffected, which indicates a certain degree of group robustness to disruptions. While this robustness is relevant, since it facilitates the modelling of the disease spread over networks (given that there is no need to predict what the new network will be after infection), this may not always be the case. In female guppies (*Poecillia reticulata*), it was found that social network structure of a group was affected differently by addition of a diseased fish as compared to addition of an uninfected fish^32^. A major difference between our experimental approach and that of Croft and colleagues^32^ is that while they formed experimental populations in artificial pools, our social groups were naturally formed and were tested in their natural environment. Their artificial populations were allowed only 24 h to acclimate, which may not have been enough to form the same type of strong relationships that we observed in our mouse population. In addition, our contact networks were based on co-localization at an important resource: a nest. While nest use is extensive and access to a nest is important for reproduction and survival in house mice^27^,^28^, a social network based on interactions outside the nest may be more vulnerable to structural changes.

Most importantly, our models demonstrate that accounting for the behavioural alteration of the diseased animals significantly reduces the projected speed and extent of disease spread within social groups. Modelling studies of human diseases are starting to take this factor into consideration with important results^37^,^38^,^39^. The observed pattern of disease spread is likely to be specific to the dense network structure of the social groups – for other network types, such as sparsely connected groups, we anticipate differing, even stronger effects of isolation of animals on the overall proportion of infected individuals.

While we have focused on disease spread, our findings can apply more broadly to other contexts, for example, transmission of information. In the same way that introduction of a disease can disrupt interactions and alter the rate of spread, the introduction of a new means of communication (e.g., social media) can change the interaction patterns of adopters (e.g. ref. 40) and alter the rate of diffusion of innovation.

In summary, our study shows that, due to behavioural alterations of infected animals, contact patterns relevant for disease transmission are not resilient to infection. Given that our model predicts that this change in behaviour significantly alters the trajectory of disease spread, this behavioural complexity should not be ignored. While an LPS injection leads to only transient changes in behaviour and physiology, many diseases cause debilitating symptoms that can last for several days. Models of disease transmission accounting for behavioural changes will be particularly relevant for infectious diseases such as Ebola or influenza, where these symptoms coincide with contagiousness.

## Methods

### Ethical note

Animal use and experimental design were approved by the Veterinary Office Zürich, Switzerland (Kantonales Veterinäramt Zürich, no. 88/2014). All experiments were carried out in accordance with the Veterinary Office Zürich guidelines and are subject to the Swiss animal protection law (TschG).

#### Study population and barn description

Since 2002, König and colleagues have followed a wild house mouse (*Mus musculus domesticus*) population living in a barn in Illnau, Switzerland (described in detail in ref. 28). Barns constitute natural habitats for this species, which is generally found living in proximity to humans. Mice can move freely in and out of the barn, constituting an open system that allows for emigration and immigration of individuals. Straw, food and water are provided weekly *ad libitum*.

Mice receive a subcutaneous implant of a radio-frequency identification (RFID) tag (Trovan ID-100, Euro ID Identifikationssysteme GmbH & Co, Germany) with a unique identity when they reach 18 g of weight. Inside the barn, there are 40 artificial nest boxes. Each box is accessible to the mice via a cylindrical tunnel, which is fitted with two round antennas that read RFID tags and allow for the determination of directional movement (i.e. entering or exiting the nest box). Information on identity of animal entering or exiting, the nest box number, and a timestamp are collected on a laptop located inside the barn. All of the information is then transferred wirelessly to a database located at the University of Zurich. Using this system (described in further detail in ref. 41), we are able to infer several different types of information, including the number of nest boxes visited by an animal, how much time each animal spent in each nest box, and which animals overlap in their visits to nest boxes.

Using antenna data collected overnight (from sunset to sunrise, when mice are most active and not disturbed by our presence), we were able to build networks describing the social contacts of the mice. We used overlap in time spent inside the nest to construct our social networks. To visualize the networks and identify separate social groups before and during the experiment we used Netdraw^42^. The social networks built revealed that the barn was composed of approximately 11 social groups during the study period.

#### Experimental procedure

The experimental manipulation consisted of injecting mice with LPS. Our purpose was to induce lethargy, a common symptom of infection. LPS induced lethargy is short lasting in adult mice (under 48 h, e.g. ref. 43). The LPS dose administered was 1.2 μg g^−1^ of body weight (~30 μg animal^−1^) based on lab studies using mice derived from this population^26^. Since LPS (LPS from E. coli, Serotype 0111:B4, Sigma-Aldrich #L4391) was dissolved in saline (Sodium Chloride solution 0.9%, Sigma-Aldrich #S8776), control injected animals received saline alone, in a volume similarly adjusted to body weight.

On the morning before visiting the barn, we would determine potential focal animals to be injected for each sex based on two criteria: we used only animals that were part of the social group (based on the social network data collected the night before the injection) and animals that did not interact with animals of another social group during that night. Sex of the focal animal and injection treatment were assigned to each experimental day and group prior to the start of the experiment. During the experiment, we visited the barn every other day, so that the frequency of our presence did not differ from normal (the barn is routinely visited several times per week to provide food and water and check for new litters). To diminish the time between capture and injection on the days we went to the barn, we used a staggered design, as follows: on a given experimental day, we did injections of mice in 3 social groups (one mouse per group), disturbed another 4 groups, and did nothing to the remainder of the groups (4 groups in this example); on the following experimental day, we did nothing to 3 social groups (previously injected), we injected mice in 4 groups (the previously disturbed groups), and we disturbed 4 groups (previously not manipulated); the rotation continued on the following experimental day. The disturbance consisted of opening the top of the artificial nest boxes, as we do routinely to inspect for new litters. The order in which each group was visited always consisted of first a disturbance, followed by injection, followed by rest, with no visit in the days in between. A social group would only receive one of these interventions in a single day. Thus, groups were only subjected to the same intervention every six days and this is the interval between two different mice within a group receiving an injection.

We arrived in the barn 2.5 h before sunset and animals were injected ~1.5 h before sunset each day. Upon arrival at the barn and setting up the necessary equipment, we first disturbed the social groups assigned to the disturbance that day and then located the focal animals. Using the information collected by the antennas on the previous night, we knew which nests the focal animals used more frequently. The capture of all focal animals averaged 24 ± 1.7 min. Focal mice IDs, sex, and weight were recorded and injections were prepared according to the weight. After releasing the injected mice near the site of capture, we made sure that there was sufficient food and water, then left immediately (~1 h before sunset).

The experiment took place between January and March 2015. We chose this time period since wild mice rarely reproduce during the winter, which meant we could minimize the effect of juveniles and pups that have not been fitted with a RFID transponder and are thus not captured in the antenna data.

At the start of the experimental period, there were a total of 257 mice registered by the antenna system. During the experimental period, a total of 19 animals were found without transponders, representing about 7.4% of the total population. In case they were at least 18 g, they received a transponder when found. A total of 38 mice were injected with LPS, 20 of which were males. A total of 39 mice were injected with saline, 20 of which were males. Mice in the population naturally die at some point. Over the course of the experiment, 20 mice were found dead, of which 2 were focal mice. One of the focal mice had been injected with saline (a male) and the other one with LPS (a female); both were removed from analysis.

#### Outline of statistical methods

The empirical analysis of the collected data contains five steps. First, we analysed whether focal mice (injected with LPS or saline) showed changes in nest box use behaviour. Subsequently, we modelled whether injected mice showed, on average, less connectivity to other mice. Results concerning the average change in behaviour were then disaggregated to determine how many of the injected mice significantly reduced contact to conspecifics. Fourth, the impact of injecting one mouse on the network structure between other members of the same group was analysed. Finally, we conducted a simulation-model to understand how the reduced connectivity of injected mice changes infection dynamics.

#### Analysis of focal individual behaviour

Statistical tests were carried out using R 3.1.2^44^. For analysis purposes, we compared antenna data obtained on the Disturbance night (hereafter “Before” time point) with data obtained on the Injection night (hereafter “Injection” time point), so that the impact of our presence was accounted for on both days. We used eight hours of antenna data beginning from sunset (~1–1.5 h post-injection) for each of those days, because a lab study demonstrated that LPS-injected mice have altered behaviours for at least this entire time period^26^. Because of the reduced amount of time used (8 h versus the initial sunset to sunrise time used to define the social groups of 13–15 h), three control injected male mice disappeared from the antenna dataset and were not part of the analysis. Accounting for these males and for the animals found dead (described above), the final sample size was 35 control (16 males) and 37 LPS (20 males) injected animals (Table S1).

To assess for alterations of individual behaviours due to injection treatment, we used change in the number of times mice entered and exited nest boxes, change in the total number of nest boxes visited, and change in the total time spent in nest boxes. Change was calculated per individual by subtracting the value obtained on Injection night by the value obtained on Disturbance night. These behaviours were analysed using linear mixed effects models (package “lme4”^45^) including a main effect of injection and sex, and the interaction between the two, and a random effect of group. When the interaction term was not significant at *P* < 0.05, it was dropped from the model and no *P* values are reported for this term in such instances. We used likelihood ratio tests to evaluate the significance of the omitted fixed terms. Visual inspection was used for assessment of fulfilment of model assumptions.

#### Analysis of social co-location networks of focal and non-focal mice

##### Data preparation

The co-location of mice within nest boxes for the 8 h following injection was recorded, including which mouse was in the nest box first. We coded complete, directed and weighted networks from this co-location data, where the direction of the tie is from the visiting to the visited animal and the tie weight denotes the time the animals spent together, summing overall visits in case of multiple encounters between mice. It should be noted that the network is not symmetric, i.e. the time mouse A visited mouse B is usually not equal to the time B visited A. Summing over both durations gives the full amount of time two mice spent together. To exclude very brief encounters in which mice left a nest box right after entering, we included only encounters that exceeded a certain duration. We adopted a 60 second threshold of minimum encounter duration in the subsequently presented analyses. However, additional analyses show that 10, 30, 60, 120 or 240 seconds of minimal encounter duration lead to the same conclusions in the statistical analyses, that is direction and significance of results from all subsequent statistical analysis were not affected by choice of thresholds, indicating robust results. This resulted in 29 complete networks, representing 29 nights, of all mice present in the barn over the experimentation period. The complete networks from all 29 nights were subsequently divided into 11–12 communities that had little or no connection to each other. These communities are the replication units of our experiment and could subsequently be categorized into groups that (i) were non-injected, (ii) had exactly one mouse that received an LPS treatment and (iii) had exactly one mouse that received a saline injection.

We analysed how a community of mice changed between two consecutive nights dependent on whether it was experimentally manipulated (one mouse injected with either LPS or control solution) or not. Communities were defined by summing the complete network over two consecutive experimental nights and finding the communities by a) disconnected components in the network and b) a clustering algorithm based on betweenness^46^ to distinguish groups that were only loosely connected by a single mouse (e.g. Fig. 1). Betweenness clustering identifies connections that are crucial in transmitting signals between different communities in a network and successively removes them until the network disaggregates into disconnected components. This resulted in approximately 300 communities by consecutive nights in which no mouse received injections of any kind on either of the nights, 35 communities over two nights that received the control injections between the first and second night, and 37 communities over two nights in which one mouse received the LPS injection between the first and second night. Of these communities, the ones that were too small (<10 mice) or too instable (<10 ties in one of the nights) for meaningful analysis were removed from the data. In the following analysis, there were 30 LPS-communities, 30 control-communities and 230 non-injected communities.

##### Statistical analysis

Statistical tests were carried out using R 3.1.2^44^, including the add-on package ‘igraph’^47^. Measures of interest (degree, in-degree and out-degree) are not normally distributed and assumptions of independence of observations are not reasonable with network data. Thus, we employed non-parametric methods to test whether the control treatment and the LPS treatment had significant effects on the measures of interest described below.

#### Average change of degree over all mice

For diseases transmitted through direct social contact, degree should function as an important predictor of infection risk in a network^48^. To test whether the average degree of focal control and LPS-injected animals decreased significantly, a distribution under the null hypothesis (that there was no change) was obtained from the data of all mice from non-injected communities. To do so, we measured the average change in degree between two days drawn from a random sample of 30 mice from all non-injected communities, resulting in one data-point of average change that could be expected if injection had no effect. This was repeated 10000 times with replacement and these 10000 values in average change constitute the distribution under the null hypothesis. The average change in degree of the LPS-group is ranked within this distribution to obtain a *p*-value, e.g. if the observed change in degree ranks the 8^th^ lowest value within the 10000 draws, a *p*-value of 0.008 is attributed. This procedure was repeated with the appropriate measures (in-degree and out-degree) for the other statistics and with the appropriate number of mice for the non-injected group. Additional analyses were carried out in which the population that formed the distribution under the null-hypothesis was not taken from all non-injected communities but from all non-injected mice from communities in which one mouse was injected. This was done to ensure that any effect was indeed due to the injection and not due to disturbance of the nest. These analyses result in the same conclusions as the previous analyses.

#### Change of degree of individual mice

The average changes in degree, in-degree and out-degree are not uniformly representing the underlying changes in degree of the individual mice. Rather, the changes are driven by some of the LPS-injected mice being isolated from their groups while the social position of other injected mice remained stable. To test which of the mice had a significant decrease in degree, in-degree and out-degree, we adopted a similar approach to the null distribution described above for groups. A distribution of expectable change of degree for each individual mouse under the null hypothesis was obtained from the mice in non-injected communities. The difference in comparison to the previous approach, where the average value of the change of 30 randomly selected mice constituted one data-point for the null-distribution, is that the relative change of one individual mouse makes up one data-point. The reason is that previously, the change in the average degree over all injected mice was compared to the average change in degree that could be expected when taking the same amount of random, non-injected mice, while here the change of an *individual* mouse and its response to the injection is under analysis. To this end, the relative change in degree of individual mice from any of the 230 non-injected groups randomly sampled with replacement was recorded to generate a distribution under the null hypothesis. In total, 4600 mice were sampled. The relative change in degree of each of the mice from the control group and the LPS group was compared to this null distribution and a *p*-value assigned. Being in the lowest 10% of the null distribution is deemed significant at α = 0.1. A significance level of 0.1 was chosen since the lowest possible *p*-value an animal can obtain is 0.06, as 6% of mice under the null distribution lose all their ties. In line with the previous section, additional analysis were carried out where the population to form the distribution under the null-hypothesis was chosen to be all non-injected mice from the communities in which one mouse was injected with LPS, to ensure that any potential effect was not due to disturbance of the mice. All results confirm the previous analysis, i.e. 40% of injected mice completely disconnect from the network.

#### Effect on social groups

To test whether the injection of a mouse results in changes in the structure of the rest of the group (excluding the focal animal), a distribution under the null-hypothesis was obtained by calculating the change between two consecutive nights in network density, network stability and average shortest path length from the 230 non-injected mice groups. Thus, for this analysis the null distribution was not obtained by sampling, but the population of non-injected networks constitute the null distribution (with *N* = 230). Network density denotes the proportion of existing ties out of all possible ties within a defined network^49^. Network stability can be measured using the Jaccard index, which calculates the proportion of ties that are present at two consecutive time-points out of all ties that were present at any of the two time points^50^. Shortest path length between two nodes is the lowest possible number of steps it takes to move from one node in the network to another node using network ties. The average shortest path length takes the mean of the shortest path length of any pair of nodes in the network. For nodes having no direct or indirect connection, the longest observed path +1 is used for calculations by convention^49^. The changes in these network metrics between the night before and after LPS injection for the networks associated with the focal animals (excluding the focal animals themselves) were compared to the null distribution.

#### Disease transmission model

To model how a proportion of infected mice becoming isolated from their social community impacts the transmission of an infectious disease, the exhibited behaviour was incorporated into an epidemiological model that is similar to the SIR model. In our model, a group of healthy animals (S) is connected by ties that differ in strength (representing time spent together from the data herein). The model is based on rounds of infection and works as follows:

1. A random animal in the network is infected with the contagious disease (now in state I).
2. A new round begins and the proportion of infected animals is recorded.
3. Each infected animal has a probability of *p*(*Iso*) to drop all its ties immediately (which is in its consequences similar to being R).
4. Each remaining infected animal can transmit the disease to all other animals it is connected to. The probability of animal *i* to infect animal *j, p*(*i* → *j*) is given by
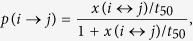
where *x*(*i* ↔ *j*) is the tie between animal *i* and animal *j*, summing over both directions of the tie, and *t*_50_ is a constant of the same unit as the tie strength that scales the probability of disease transmission. Following the equation, transmission probability has an s-shaped form with a 50% probability occurring at *t*_50_. For example, given *t*_50_ = 300, there is a 50% probability of animal *i* to transmit the disease to animal *j* if they spent 300 seconds together during the experimentation period, if they spent 150 seconds together the transmission probability is 33%, and if they spent 600 seconds together, *p*(*i* → *j*) = 66%.
5. After all potential transmissions are evaluated and executed accordingly, all animals that were infected before the beginning of the current round are no longer contagious and are moved to state R.
6. Unless all animals are in state S or R, a new round is started (step 2).

The presented model was tested with *p*(*Iso*) of 0%, 10%, 20%, 30%, 40% (empirically observed), and 50%. The networks used for the simulations are all 230 networks of non-injected mice, each one starting with 6 different, randomly chosen mice. Thus, Fig. 3a,b are based on 1380 simulations for each scenario. In each simulation *t*_50_ = 600, while the results are qualitatively robust to other values of 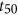.

### Data accessibility

Data and R code for the simulation model are deposited in the Dryad repository (doi:10.5061/dryad.nk1b8).

## Additional Information

**How to cite this article**: Lopes, P. C. *et al*. Infection-induced behavioural changes reduce connectivity and the potential for disease spread in wild mice contact networks. *Sci. Rep.* **6**, 31790; doi: 10.1038/srep31790 (2016).

## Supplementary Material

Supplementary Information

## Figures and Tables

**Table 1:** Average change in social contacts over all mice.

Variable	LPS-injected		Control-injected	
	Average change (∆)	*P*	Average change (∆)	*P*
Degree	**−4.8**	**0.008**	−1.8	0.19
In-degree	−1.6	0.06	−1.4	0.10
Out-degree	**−3.2**	**0.001**	−0.5	0.35
Time in social contact (sec)	**−19180.0**	**0.015**	−11338.9	0.099
Time visited (sec)	−6619.7	0.105	−3699.63	0.24
Time visiting (sec)	**−12560.3**	**0.0063**	−7639.3	0.065
Number of interactions	**−61.15**	**0.018**	−13.2	0.30
Number of visits by others	−9.29	0.25	−0.13	0.506
Number of visits to others	**−51.85**	**0.015**	−13.07	0.27

A *P*-value was calculated by comparing the average change in injected mice to a distribution of average change obtained by repeated draws (10,000) of equal sized groups of randomly selected mice. Statistically significant differences at *P* < 0.05 are highlighted in bold.
