# Supplementary material for: Infection-induced behavioural changes reduce connectivity and the potential for disease spread in wild mice contact networks
Source: Sci Rep. 2016 Aug 22;6:31790. doi: 10.1038/srep31790 (PMC4993150; doi:10.1038/srep31790)
Supplement: Supplementary Information [file srep31790-s1.pdf]

Supplementary information to:

Infection-induced behavioural changes reduce connectivity and the potential for disease spread in wild mice contact networks

Patricia C. Lopes[1]*, Per Block[2], Barbara König[1]

[1]Department of Evolutionary Biology and Environmental Studies, University of Zurich, Zurich, Switzerland.

[2]Department of Humanities, Social and Political Sciences, ETH, Zurich, Switzerland.

*Correspondence to: patricia.lopes@ieu.uzh.ch.

Supplementary Information

Table S1. Final number of injected animals per social group.

group	sex	injection	# of animals
c2a	Fem	Control	1
	Fem	LPS	2
	Male	Control	3
	Male	LPS	2
a1	Fem	Control	2
	Fem	LPS	1
	Male	Control	2
	Male	LPS	1
a6	Fem	Control	2
	Fem	LPS	2
	Male	Control	1
	Male	LPS	1
a7	Fem	Control	2
	Fem	LPS	1
	Male	Control	2
	Male	LPS	2
a9	Fem	Control	2
	Fem	LPS	2
	Male	Control	1
	Male	LPS	2
b4	Fem	Control	2
	Fem	LPS	1
	Male	Control	1
	Male	LPS	3
b5	Fem	Control	2
	Fem	LPS	1
	Male	Control	2
	Male	LPS	2
b8	Fem	Control	1
	Fem	LPS	2
	Male	Control	1
	Male	LPS	2
c10	Fem	Control	1
	Fem	LPS	1
	Male	Control	1
	Male	LPS	2
c2b	Fem	Control	2
	Fem	LPS	2
	Male	Control	2
	Male	LPS	1
c3	Fem	Control	2
	Fem	LPS	2
	Male	LPS	2

Figure S1. Change in number of social partners per focal mouse. Change in degree (a), out-degree (b) and in-degree (c) of individual mice injected with either LPS or control on the night of injection or two nights before, assessed by overlap in nest box use overnight. Each line represents one mouse. Relative to the null distribution, solid lines show a significant decrease in measure, dotted lines no significant change, and dashed lines a significant increase.

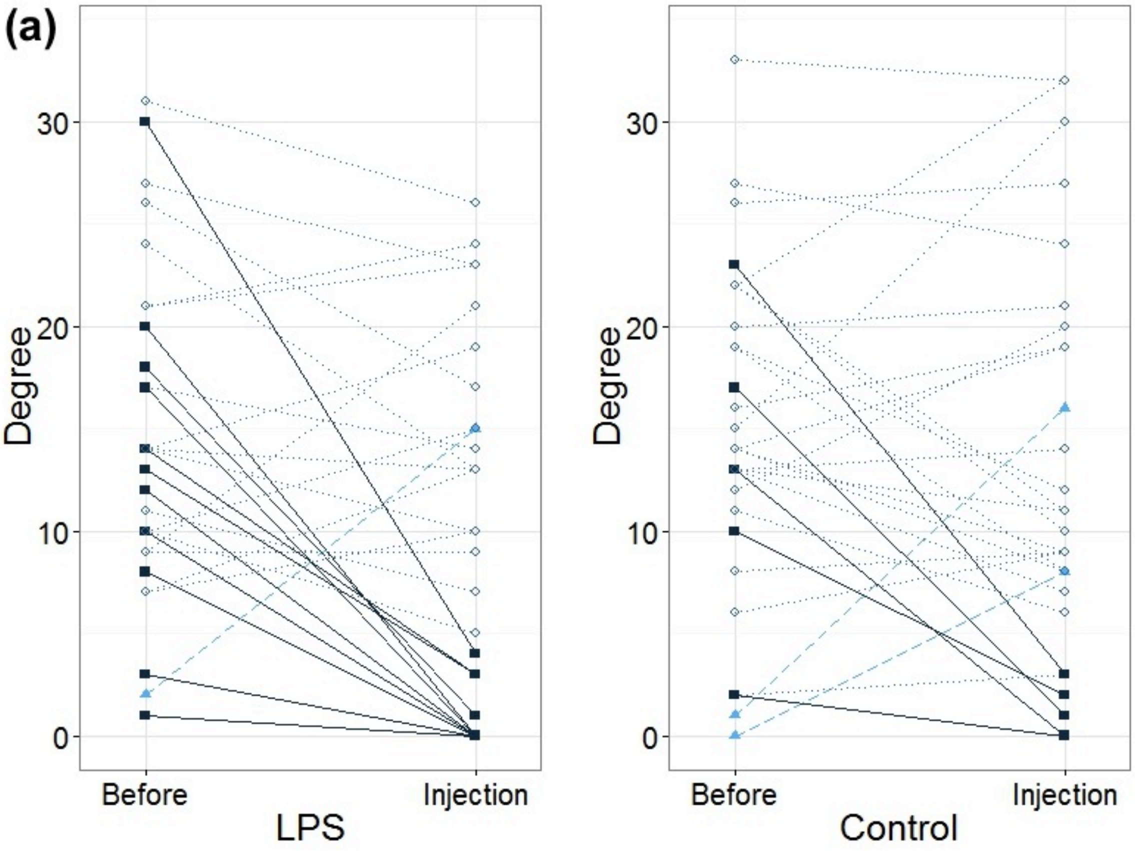

(a)
Degree
30
20
10
0
Before
Injection
LPS
Degree
30
20
10
0
Before
Injection
Control

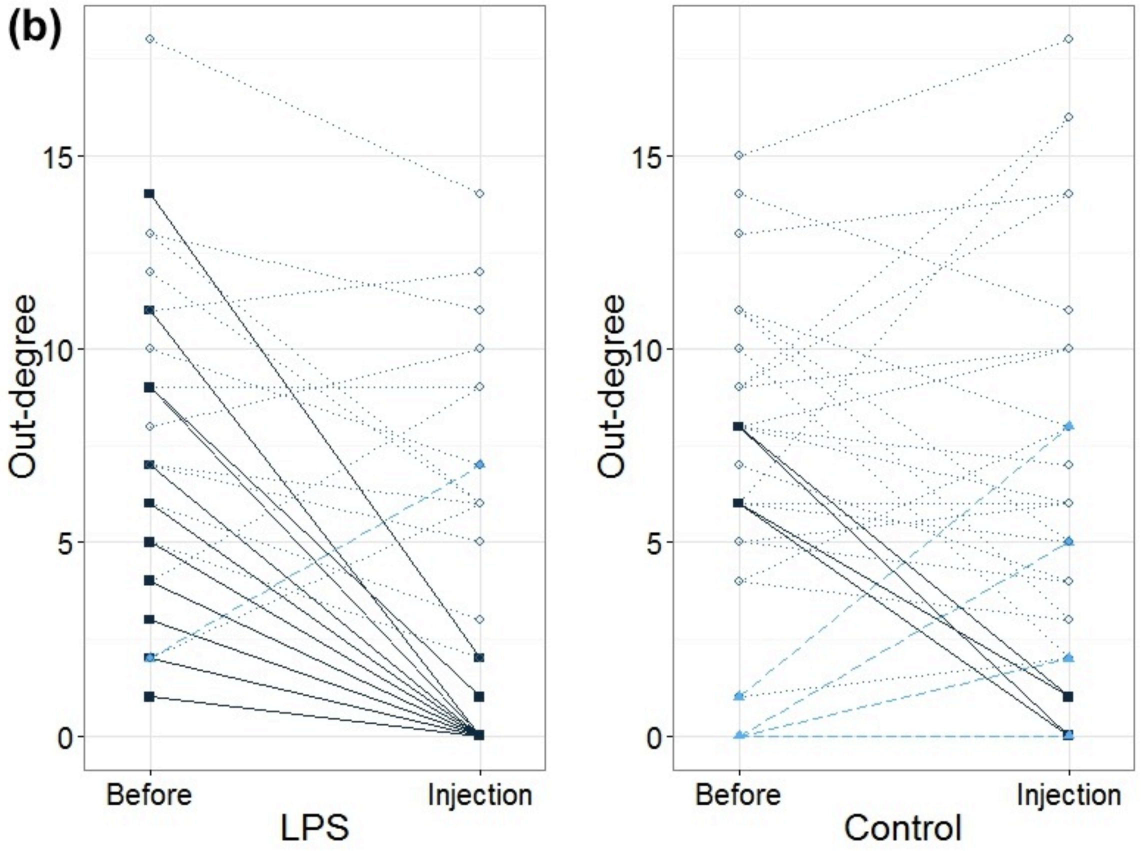
(b)
Out-degree
15
10
5
0
Before
Injection
LPS
Out-degree
15
10
5
0
Before
Injection
Control

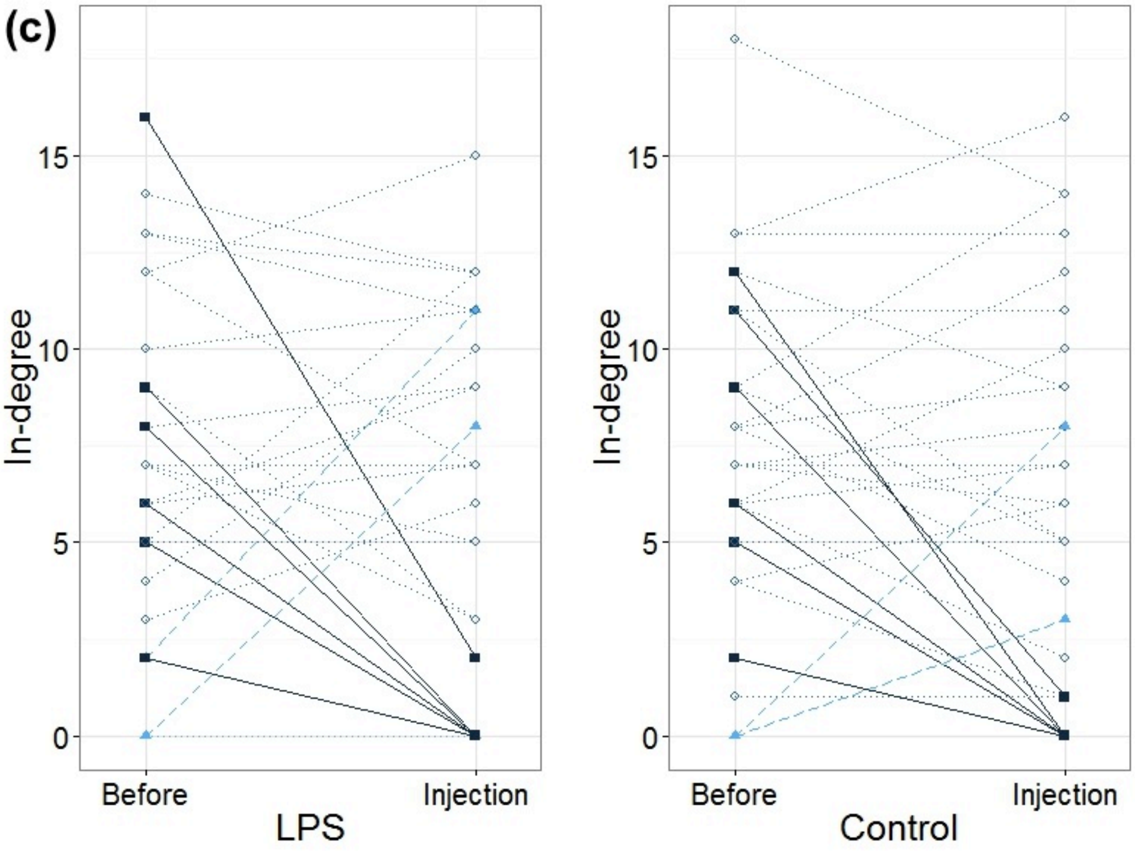
(c)
In-degree
0
5
10
15
Before
Injection
LPS
In-degree
0
5
10
15
Before
Injection
Control
